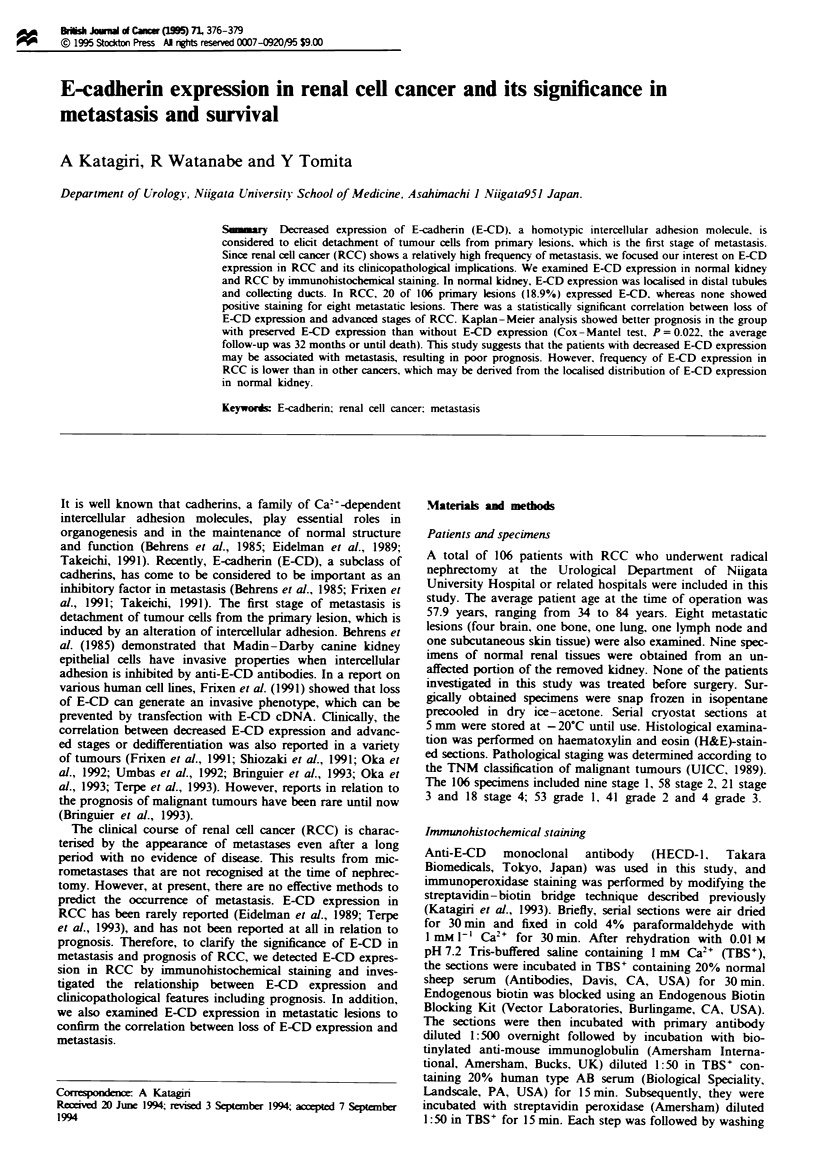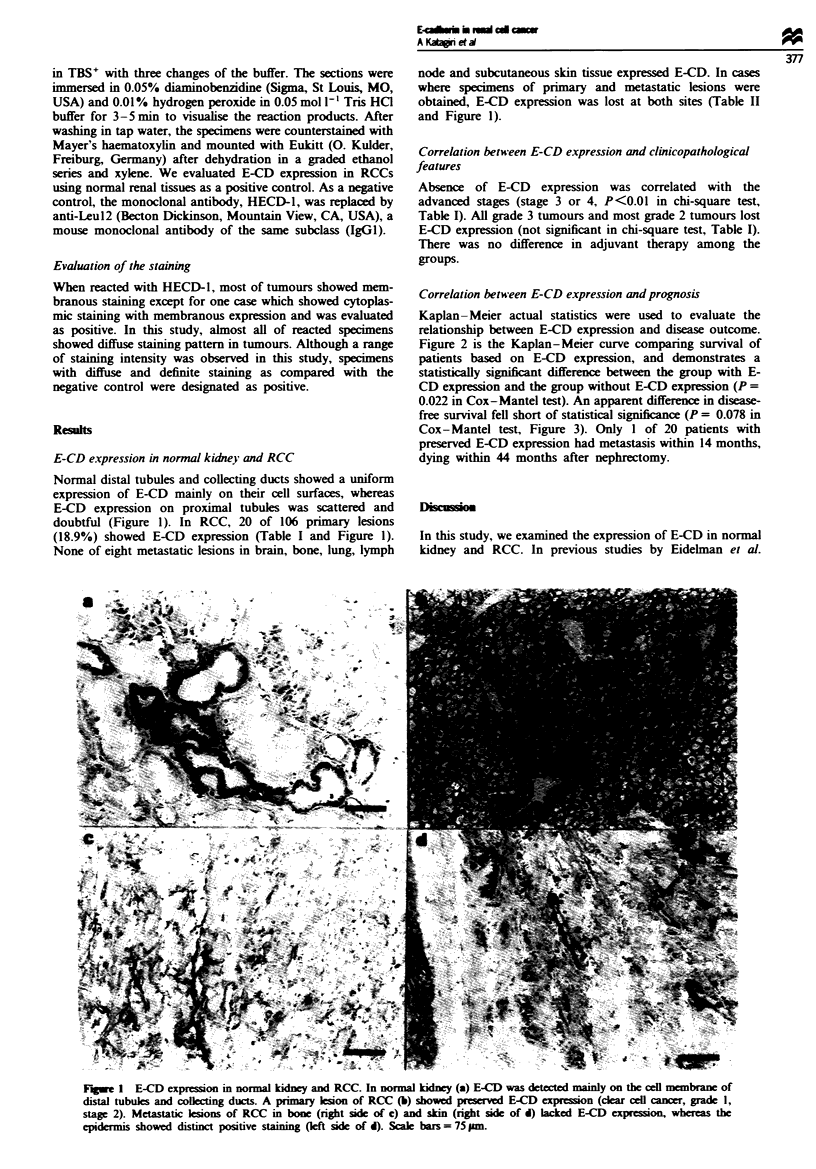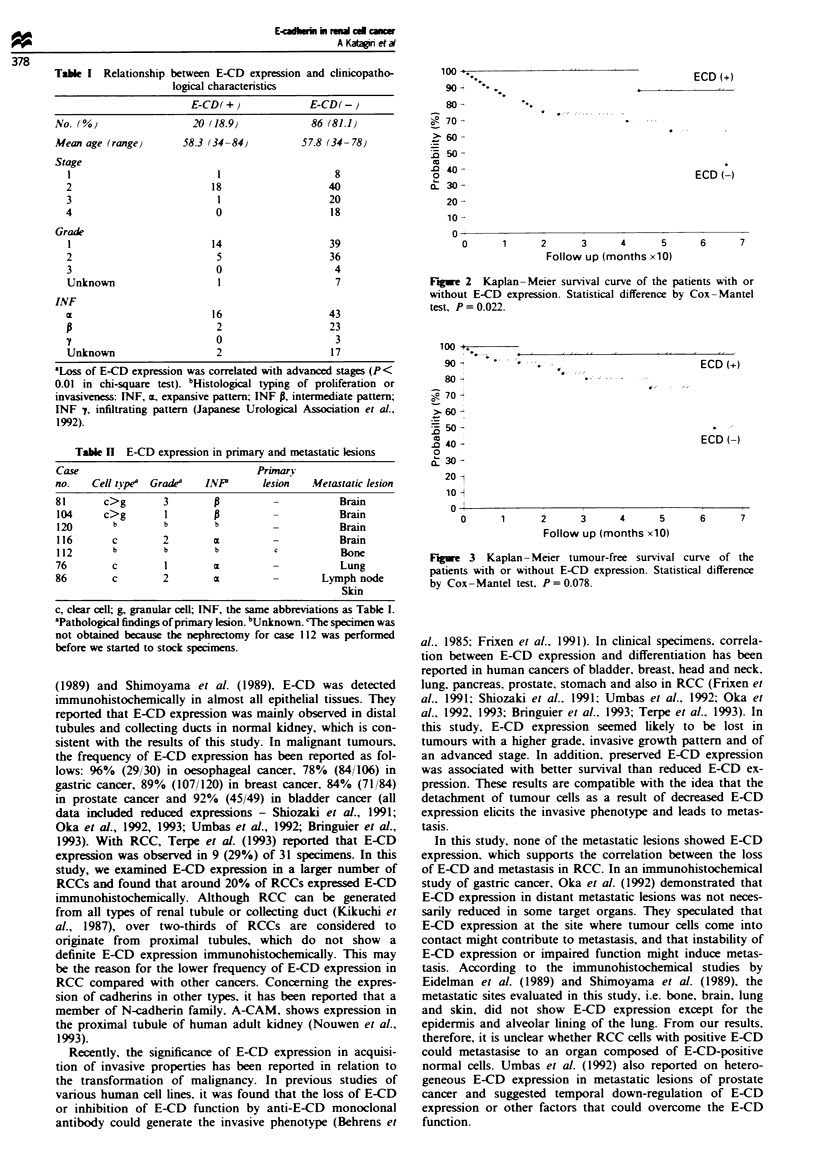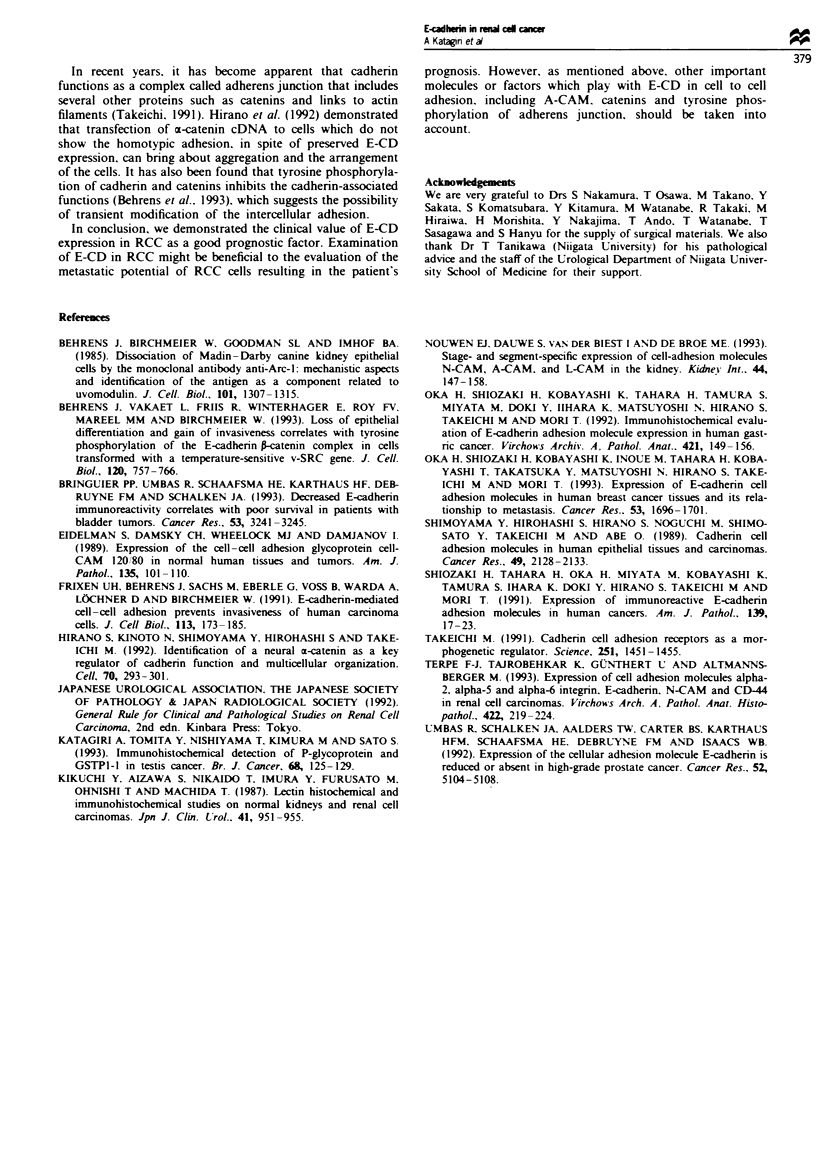# E-cadherin expression in renal cell cancer and its significance in metastasis and survival.

**DOI:** 10.1038/bjc.1995.76

**Published:** 1995-02

**Authors:** A. Katagiri, R. Watanabe, Y. Tomita

**Affiliations:** Department of Urology, Niigata University School of Medicine, Japan.

## Abstract

**Images:**


British Journal of Cancer (1995) 71, 376-379

© 1995 Stockton Press All rights reserved 0007-0920/95 $9.00

### E-cadherin expression in renal cell cancer and its significance in metastasis and survival

A Katagiri, R Watanabe and Y Tomita

*Department of Urology, Niigata University School of Medicine, Asahimachi 1 Niigata951 Japan.*

**Summary** Decreased expression of E-cadherin (E-CD), a homotypic intercellular adhesion molecule, is considered to elicit detachment of tumour cells from primary lesions, which is the first stage of metastasis. Since renal cell cancer (RCC) shows a relatively high frequency of metastasis, we focused our interest on E-CD expression in RCC and its clinicopathological implications. We examined E-CD expression in normal kidney and RCC by immunohistochemical staining. In normal kidney, E-CD expression was localised in distal tubules and collecting ducts. In RCC, 20 of 106 primary lesions (18.9%) expressed E-CD, whereas none showed positive staining for eight metastatic lesions. There was a statistically significant correlation between loss of E-CD expression and advanced stages of RCC. Kaplan-Meier analysis showed better prognosis in the group with preserved E-CD expression than without E-CD expression (Cox-Mantel test, $P = 0.022$, the average follow-up was 32 months or until death). This study suggests that the patients with decreased E-CD expression may be associated with metastasis, resulting in poor prognosis. However, frequency of E-CD expression in RCC is lower than in other cancers, which may be derived from the localised distribution of E-CD expression in normal kidney.

**Keywords:** E-cadherin; renal cell cancer; metastasis

It is well known that cadherins, a family of $Ca^{2+}$-dependent intercellular adhesion molecules, play essential roles in organogenesis and in the maintenance of normal structure and function (Behrens *et al.*, 1985; Eidelman *et al.*, 1989; Takeichi, 1991). Recently, E-cadherin (E-CD), a subclass of cadherins, has come to be considered to be important as an inhibitory factor in metastasis (Behrens *et al.*, 1985; Frixen *et al.*, 1991; Takeichi, 1991). The first stage of metastasis is detachment of tumour cells from the primary lesion, which is induced by an alteration of intercellular adhesion. Behrens *et al.* (1985) demonstrated that Madin-Darby canine kidney epithelial cells have invasive properties when intercellular adhesion is inhibited by anti-E-CD antibodies. In a report on various human cell lines, Frixen *et al.* (1991) showed that loss of E-CD can generate an invasive phenotype, which can be prevented by transfection with E-CD cDNA. Clinically, the correlation between decreased E-CD expression and advanced stages or dedifferentiation was also reported in a variety of tumours (Frixen *et al.*, 1991; Shiozaki *et al.*, 1991; Oka *et al.*, 1992; Umbas *et al.*, 1992; Bringuier *et al.*, 1993; Oka *et al.*, 1993; Terpe *et al.*, 1993). However, reports in relation to the prognosis of malignant tumours have been rare until now (Bringuier *et al.*, 1993).

The clinical course of renal cell cancer (RCC) is characterised by the appearance of metastases even after a long period with no evidence of disease. This results from micrometastases that are not recognised at the time of nephrectomy. However, at present, there are no effective methods to predict the occurrence of metastasis. E-CD expression in RCC has been rarely reported (Eidelman *et al.*, 1989; Terpe *et al.*, 1993), and has not been reported at all in relation to prognosis. Therefore, to clarify the significance of E-CD in metastasis and prognosis of RCC, we detected E-CD expression in RCC by immunohistochemical staining and investigated the relationship between E-CD expression and clinicopathological features including prognosis. In addition, we also examined E-CD expression in metastatic lesions to confirm the correlation between loss of E-CD expression and metastasis.

Correspondence: A Katagiri
Received 20 June 1994; revised 3 September 1994; accepted 7 September 1994

### Materials and methods

### *Patients and specimens*

A total of 106 patients with RCC who underwent radical nephrectomy at the Urological Department of Niigata University Hospital or related hospitals were included in this study. The average patient age at the time of operation was 57.9 years, ranging from 34 to 84 years. Eight metastatic lesions (four brain, one bone, one lung, one lymph node and one subcutaneous skin tissue) were also examined. Nine specimens of normal renal tissues were obtained from an unaffected portion of the removed kidney. None of the patients investigated in this study was treated before surgery. Surgically obtained specimens were snap frozen in isopentane precooled in dry ice-acetone. Serial cryostat sections at 5 mm were stored at −20°C until use. Histological examination was performed on haematoxylin and eosin (H&E)-stained sections. Pathological staging was determined according to the TNM classification of malignant tumours (UICC, 1989). The 106 specimens included nine stage 1, 58 stage 2, 21 stage 3 and 18 stage 4; 53 grade 1, 41 grade 2 and 4 grade 3.

### *Immunohistochemical staining*

Anti-E-CD monoclonal antibody (HECD-1, Takara Biomedicals, Tokyo, Japan) was used in this study, and immunoperoxidase staining was performed by modifying the streptavidin-biotin bridge technique described previously (Katagiri *et al.*, 1993). Briefly, serial sections were air dried for 30 min and fixed in cold 4% paraformaldehyde with 1 mM $l^{-1}$ $Ca^{2+}$ for 30 min. After rehydration with 0.01 M pH 7.2 Tris-buffered saline containing 1 mM $Ca^{2+}$ ($TBS^+$), the sections were incubated in $TBS^+$ containing 20% normal sheep serum (Antibodies, Davis, CA, USA) for 30 min. Endogenous biotin was blocked using an Endogenous Biotin Blocking Kit (Vector Laboratories, Burlingame, CA, USA). The sections were then incubated with primary antibody diluted 1:500 overnight followed by incubation with biotinylated anti-mouse immunoglobulin (Amersham International, Amersham, Bucks, UK) diluted 1:50 in $TBS^+$ containing 20% human type AB serum (Biological Speciality, Landscale, PA, USA) for 15 min. Subsequently, they were incubated with streptavidin peroxidase (Amersham) diluted 1:50 in $TBS^+$ for 15 min. Each step was followed by washing in TBS+ with three changes of the buffer. The sections were immersed in 0.05% diaminobenzidine (Sigma, St Louis, MO, USA) and 0.01% hydrogen peroxide in 0.05 mol $l^{-1}$ Tris HCl buffer for 3–5 min to visualise the reaction products. After washing in tap water, the specimens were counterstained with Mayer's haematoxylin and mounted with Eukitt (O. Kulder, Freiburg, Germany) after dehydration in a graded ethanol series and xylene. We evaluated E-CD expression in RCCs using normal renal tissues as a positive control. As a negative control, the monoclonal antibody, HECD-1, was replaced by anti-Leu12 (Becton Dickinson, Mountain View, CA, USA), a mouse monoclonal antibody of the same subclass (IgG1).

*Evaluation of the staining*

When reacted with HECD-1, most of tumours showed membranous staining except for one case which showed cytoplasmic staining with membranous expression and was evaluated as positive. In this study, almost all of reacted specimens showed diffuse staining pattern in tumours. Although a range of staining intensity was observed in this study, specimens with diffuse and definite staining as compared with the negative control were designated as positive.

### Results

*E-CD expression in normal kidney and RCC*

Normal distal tubules and collecting ducts showed a uniform expression of E-CD mainly on their cell surfaces, whereas E-CD expression on proximal tubules was scattered and doubtful (Figure 1). In RCC, 20 of 106 primary lesions (18.9%) showed E-CD expression (Table I and Figure 1). None of eight metastatic lesions in brain, bone, lung, lymph node and subcutaneous skin tissue expressed E-CD. In cases where specimens of primary and metastatic lesions were obtained, E-CD expression was lost at both sites (Table II and Figure 1).

*Correlation between E-CD expression and clinicopathological features*

Absence of E-CD expression was correlated with the advanced stages (stage 3 or 4, $P<0.01$ in chi-square test, Table I). All grade 3 tumours and most grade 2 tumours lost E-CD expression (not significant in chi-square test, Table I). There was no difference in adjuvant therapy among the groups.

*Correlation between E-CD expression and prognosis*

Kaplan–Meier actual statistics were used to evaluate the relationship between E-CD expression and disease outcome. Figure 2 is the Kaplan–Meier curve comparing survival of patients based on E-CD expression, and demonstrates a statistically significant difference between the group with E-CD expression and the group without E-CD expression ($P=0.022$ in Cox–Mantel test). An apparent difference in disease-free survival fell short of statistical significance ($P=0.078$ in Cox–Mantel test, Figure 3). Only 1 of 20 patients with preserved E-CD expression had metastasis within 14 months, dying within 44 months after nephrectomy.

### Discussion

In this study, we examined the expression of E-CD in normal kidney and RCC. In previous studies by Eidelman *et al.*

**Figure 1** E-CD expression in normal kidney and RCC. In normal kidney (**a**) E-CD was detected mainly on the cell membrane of distal tubules and collecting ducts. A primary lesion of RCC (**b**) showed preserved E-CD expression (clear cell cancer, grade 1, stage 2). Metastatic lesions of RCC in bone (right side of **c**) and skin (right side of **d**) lacked E-CD expression, whereas the epidermis showed distinct positive staining (left side of **d**). Scale bars = 75 μm.

**Table I** Relationship between E-CD expression and clinicopathological characteristics

| | E-CD(+) | E-CD(−) |
|---|---|---|
| *No. (%)* | *20 (18.9)* | *86 (81.1)* |
| *Mean age (range)* | *58.3 (34–84)* | *57.8 (34–78)* |
| *Stage* | | |
| 1 | 1 | 8 |
| 2 | 18 | 40 |
| 3 | 1 | 20 |
| 4 | 0 | 18 |
| *Grade* | | |
| 1 | 14 | 39 |
| 2 | 5 | 36 |
| 3 | 0 | 4 |
| Unknown | 1 | 7 |
| *INF* | | |
| α | 16 | 43 |
| β | 2 | 23 |
| γ | 0 | 3 |
| Unknown | 2 | 17 |

[a]Loss of E-CD expression was correlated with advanced stages ($P < 0.01$ in chi-square test). [b]Histological typing of proliferation or invasiveness: INF, α, expansive pattern; INF β, intermediate pattern; INF γ, infiltrating pattern (Japanese Urological Association *et al.*, 1992).

**Table II** E-CD expression in primary and metastatic lesions

| Case no. | Cell type[a] | Grade[a] | INF[a] | Primary lesion | Metastatic lesion |
|---|---|---|---|---|---|
| 81 | c>g | 3 | β | − | Brain |
| 104 | c>g | 1 | β | − | Brain |
| 120 | [b] | [b] | [b] | − | Brain |
| 116 | c | 2 | α | − | Brain |
| 112 | [b] | [b] | [b] | [c] | Bone |
| 76 | c | 1 | α | − | Lung |
| 86 | c | 2 | α | − | Lymph node Skin |

c, clear cell; g, granular cell; INF, the same abbreviations as Table I. [a]Pathological findings of primary lesion. [b]Unknown. [c]The specimen was not obtained because the nephrectomy for case 112 was performed before we started to stock specimens.

**Figure 2** Kaplan–Meier survival curve of the patients with or without E-CD expression. Statistical difference by Cox–Mantel test, $P = 0.022$.

**Figure 3** Kaplan–Meier tumour-free survival curve of the patients with or without E-CD expression. Statistical difference by Cox–Mantel test, $P = 0.078$.

(1989) and Shimoyama *et al.* (1989), E-CD was detected immunohistochemically in almost all epithelial tissues. They reported that E-CD expression was mainly observed in distal tubules and collecting ducts in normal kidney, which is consistent with the results of this study. In malignant tumours, the frequency of E-CD expression has been reported as follows: 96% (29/30) in oesophageal cancer, 78% (84/106) in gastric cancer, 89% (107/120) in breast cancer, 84% (71/84) in prostate cancer and 92% (45/49) in bladder cancer (all data included reduced expressions – Shiozaki *et al.*, 1991; Oka *et al.*, 1992, 1993; Umbas *et al.*, 1992; Bringuier *et al.*, 1993). With RCC, Terpe *et al.* (1993) reported that E-CD expression was observed in 9 (29%) of 31 specimens. In this study, we examined E-CD expression in a larger number of RCCs and found that around 20% of RCCs expressed E-CD immunohistochemically. Although RCC can be generated from all types of renal tubule or collecting duct (Kikuchi *et al.*, 1987), over two-thirds of RCCs are considered to originate from proximal tubules, which do not show a definite E-CD expression immunohistochemically. This may be the reason for the lower frequency of E-CD expression in RCC compared with other cancers. Concerning the expression of cadherins in other types, it has been reported that a member of N-cadherin family, A-CAM, shows expression in the proximal tubule of human adult kidney (Nouwen *et al.*, 1993).

Recently, the significance of E-CD expression in acquisition of invasive properties has been reported in relation to the transformation of malignancy. In previous studies of various human cell lines, it was found that the loss of E-CD or inhibition of E-CD function by anti-E-CD monoclonal antibody could generate the invasive phenotype (Behrens *et al.*, 1985; Frixen *et al.*, 1991). In clinical specimens, correlation between E-CD expression and differentiation has been reported in human cancers of bladder, breast, head and neck, lung, pancreas, prostate, stomach and also in RCC (Frixen *et al.*, 1991; Shiozaki *et al.*, 1991; Umbas *et al.*, 1992; Oka *et al.*, 1992, 1993; Bringuier *et al.*, 1993; Terpe *et al.*, 1993). In this study, E-CD expression seemed likely to be lost in tumours with a higher grade, invasive growth pattern and of an advanced stage. In addition, preserved E-CD expression was associated with better survival than reduced E-CD expression. These results are compatible with the idea that the detachment of tumour cells as a result of decreased E-CD expression elicits the invasive phenotype and leads to metastasis.

In this study, none of the metastatic lesions showed E-CD expression, which supports the correlation between the loss of E-CD and metastasis in RCC. In an immunohistochemical study of gastric cancer, Oka *et al.* (1992) demonstrated that E-CD expression in distant metastatic lesions was not necessarily reduced in some target organs. They speculated that E-CD expression at the site where tumour cells come into contact might contribute to metastasis, and that instability of E-CD expression or impaired function might induce metastasis. According to the immunohistochemical studies by Eidelman *et al.* (1989) and Shimoyama *et al.* (1989), the metastatic sites evaluated in this study, i.e. bone, brain, lung and skin, did not show E-CD expression except for the epidermis and alveolar lining of the lung. From our results, therefore, it is unclear whether RCC cells with positive E-CD could metastasise to an organ composed of E-CD-positive normal cells. Umbas *et al.* (1992) also reported on heterogeneous E-CD expression in metastatic lesions of prostate cancer and suggested temporal down-regulation of E-CD expression or other factors that could overcome the E-CD function.

In recent years, it has become apparent that cadherin functions as a complex called adherens junction that includes several other proteins such as catenins and links to actin filaments (Takeichi, 1991). Hirano *et al.* (1992) demonstrated that transfection of α-catenin cDNA to cells which do not show the homotypic adhesion, in spite of preserved E-CD expression, can bring about aggregation and the arrangement of the cells. It has also been found that tyrosine phosphorylation of cadherin and catenins inhibits the cadherin-associated functions (Behrens *et al.*, 1993), which suggests the possibility of transient modification of the intercellular adhesion.

In conclusion, we demonstrated the clinical value of E-CD expression in RCC as a good prognostic factor. Examination of E-CD in RCC might be beneficial to the evaluation of the metastatic potential of RCC cells resulting in the patient's prognosis. However, as mentioned above, other important molecules or factors which play with E-CD in cell to cell adhesion, including A-CAM, catenins and tyrosine phosphorylation of adherens junction, should be taken into account.

**Acknowledgements**

We are very grateful to Drs S Nakamura, T Osawa, M Takano, Y Sakata, S Komatsubara, Y Kitamura, M Watanabe, R Takaki, M Hiraiwa, H Morishita, Y Nakajima, T Ando, T Watanabe, T Sasagawa and S Hanyu for the supply of surgical materials. We also thank Dr T Tanikawa (Niigata University) for his pathological advice and the staff of the Urological Department of Niigata University School of Medicine for their support.

**References**

BEHRENS J, BIRCHMEIER W, GOODMAN SL AND IMHOF BA. (1985). Dissociation of Madin–Darby canine kidney epithelial cells by the monoclonal antibody anti-Arc-1: mechanistic aspects and identification of the antigen as a component related to uvomodulin. *J. Cell. Biol.*, **101**, 1307–1315.

BEHRENS J, VAKAET L, FRIIS R, WINTERHAGER E, ROY FV, MAREEL MM AND BIRCHMEIER W. (1993). Loss of epithelial differentiation and gain of invasiveness correlates with tyrosine phosphorylation of the E-cadherin/β-catenin complex in cells transformed with a temperature-sensitive v-SRC gene. *J. Cell. Biol.*, **120**, 757–766.

BRINGUIER PP, UMBAS R, SCHAAFSMA HE, KARTHAUS HF, DEBRUYNE FM AND SCHALKEN JA. (1993). Decreased E-cadherin immunoreactivity correlates with poor survival in patients with bladder tumors. *Cancer Res.*, **53**, 3241–3245.

EIDELMAN S, DAMSKY CH, WHEELOCK MJ AND DAMJANOV I. (1989). Expression of the cell–cell adhesion glycoprotein cell-CAM 120/80 in normal human tissues and tumors. *Am. J. Pathol.*, **135**, 101–110.

FRIXEN UH, BEHRENS J, SACHS M, EBERLE G, VOSS B, WARDA A, LÖCHNER D AND BIRCHMEIER W. (1991). E-cadherin-mediated cell–cell adhesion prevents invasiveness of human carcinoma cells. *J. Cell Biol.*, **113**, 173–185.

HIRANO S, KINOTO N, SHIMOYAMA Y, HIROHASHI S AND TAKEICHI M. (1992). Identification of a neural α-catenin as a key regulator of cadherin function and multicellular organization. *Cell*, **70**, 293–301.

JAPANESE UROLOGICAL ASSOCIATION, THE JAPANESE SOCIETY OF PATHOLOGY & JAPAN RADIOLOGICAL SOCIETY (1992). *General Rule for Clinical and Pathological Studies on Renal Cell Carcinoma*, 2nd edn. Kinbara Press: Tokyo.

KATAGIRI A, TOMITA Y, NISHIYAMA T, KIMURA M AND SATO S. (1993). Immunohistochemical detection of P-glycoprotein and GSTP1-1 in testis cancer. *Br. J. Cancer*, **68**, 125–129.

KIKUCHI Y, AIZAWA S, NIKAIDO T, IMURA Y, FURUSATO M, OHNISHI T AND MACHIDA T. (1987). Lectin histochemical and immunohistochemical studies on normal kidneys and renal cell carcinomas. *Jpn J. Clin. Urol.*, **41**, 951–955.

NOUWEN EJ, DAUWE S, VAN DER BIEST I AND DE BROE ME. (1993). Stage- and segment-specific expression of cell-adhesion molecules N-CAM, A-CAM, and L-CAM in the kidney. *Kidney Int.*, **44**, 147–158.

OKA H, SHIOZAKI H, KOBAYASHI K, TAHARA H, TAMURA S, MIYATA M, DOKI Y, IIHARA K, MATSUYOSHI N, HIRANO S, TAKEICHI M AND MORI T. (1992). Immunohistochemical evaluation of E-cadherin adhesion molecule expression in human gastric cancer. *Virchows Archiv. A, Pathol. Anat.*, **421**, 149–156.

OKA H, SHIOZAKI H, KOBAYASHI K, INOUE M, TAHARA H, KOBAYASHI T, TAKATSUKA Y, MATSUYOSHI N, HIRANO S, TAKEICHI M AND MORI T. (1993). Expression of E-cadherin cell adhesion molecules in human breast cancer tissues and its relationship to metastasis. *Cancer Res.*, **53**, 1696–1701.

SHIMOYAMA Y, HIROHASHI S, HIRANO S, NOGUCHI M, SHIMOSATO Y, TAKEICHI M AND ABE O. (1989). Cadherin cell adhesion molecules in human epithelial tissues and carcinomas. *Cancer Res.*, **49**, 2128–2133.

SHIOZAKI H, TAHARA H, OKA H, MIYATA M, KOBAYASHI K, TAMURA S, IHARA K, DOKI Y, HIRANO S, TAKEICHI M AND MORI T. (1991). Expression of immunoreactive E-cadherin adhesion molecules in human cancers. *Am. J. Pathol.*, **139**, 17–23.

TAKEICHI M. (1991). Cadherin cell adhesion receptors as a morphogenetic regulator. *Science*, **251**, 1451–1455.

TERPE F-J, TAJROBEHKAR K, GÜNTHERT U AND ALTMANNSBERGER M. (1993). Expression of cell adhesion molecules alpha-2, alpha-5 and alpha-6 integrin, E-cadherin, N-CAM and CD-44 in renal cell carcinomas. *Virchows Arch. A, Pathol. Anat. Histopathol.*, **422**, 219–224.

UMBAS R, SCHALKEN JA, AALDERS TW, CARTER BS, KARTHAUS HFM, SCHAAFSMA HE, DEBRUYNE FM AND ISAACS WB. (1992). Expression of the cellular adhesion molecule E-cadherin is reduced or absent in high-grade prostate cancer. *Cancer Res.*, **52**, 5104–5108.